# Multidisciplinary approach to improving documentation of visual acuity in patients presenting with ocular trauma

**DOI:** 10.1186/cc12203

**Published:** 2013-03-19

**Authors:** L Low, M Johnston

**Affiliations:** 1 NHS Tayside, Dundee, UK

## Introduction

This study aimed to implement a multidisciplinary quality improvement project in Perth Royal Infirmary A&E department to improve documentation of visual acuity (VA) in patients presenting with ocular injury.

## Methods

The improvement project involves a three-pronged multidisciplinary approach: ensure that equipment required for VA testing (Snellen chart and pinhole mask) was readily available; encourage VA testing at first point of contact with A&E staff, both nursing/medical staff; and refresher online course on how to test for VA quickly and accurately, in the 6/X format. We compared the pre-intervention (2 September to 2 October 2012) and post-intervention (11 October to 19 November 2012) rates of VA documentation using the chi-square test.

## Results

**Figure 1:**
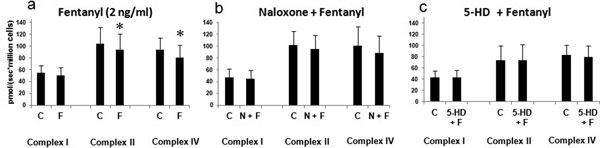


During the pre-intervention period, of a total of 44 patients who presented to A&E with eye injury, only 36 patients (95%) had their VA tested. Following intervention, there was significant improvement in VA testing, where all 43 patients presenting to A&E with eye injuries had their VA tested (100%, *P *= 0.02). Documentation of VA in the correct (6/X) format increased from 82 to 84% following intervention. There was a 15% improvement in documentation of best-corrected VA, from 48 to 63% post intervention. See Figure 1.

## Conclusion

Through a multidisciplinary approach, we were successful in achieving our target of 100% VA documentation rate in all patients presenting with eye injury to PRI A&E.

